# Supplementary material for: The Cellular Abundance of Chemoreceptors, Chemosensory Signaling Proteins, Sensor Histidine Kinases, and Solute Binding Proteins of Pseudomonas aeruginosa Provides Insight into Sensory Preferences and Signaling Mechanisms
Source: Int J Mol Sci. 2023 Jan 10;24(2):1363. doi: 10.3390/ijms24021363 (PMC9864684; doi:10.3390/ijms24021363)
Supplement: Supplementary file 1 [file ijms-24-01363-s001.zip › Supplementary Figures S1-S2.pdf]

Supplementary Material

to

The Cellular Abundance of Chemoreceptors, Chemosensory Signaling Proteins, Sensor Histidine Kinases, and Solute Binding Proteins of *Pseudomonas aeruginosa* Provides Insight into Sensory Preferences and Signaling Mechanisms

by

Miguel A. Matilla, Roberta Genova, David Martín-Mora, Sandra Maaβ, Dörte Becher and Tino Krell *

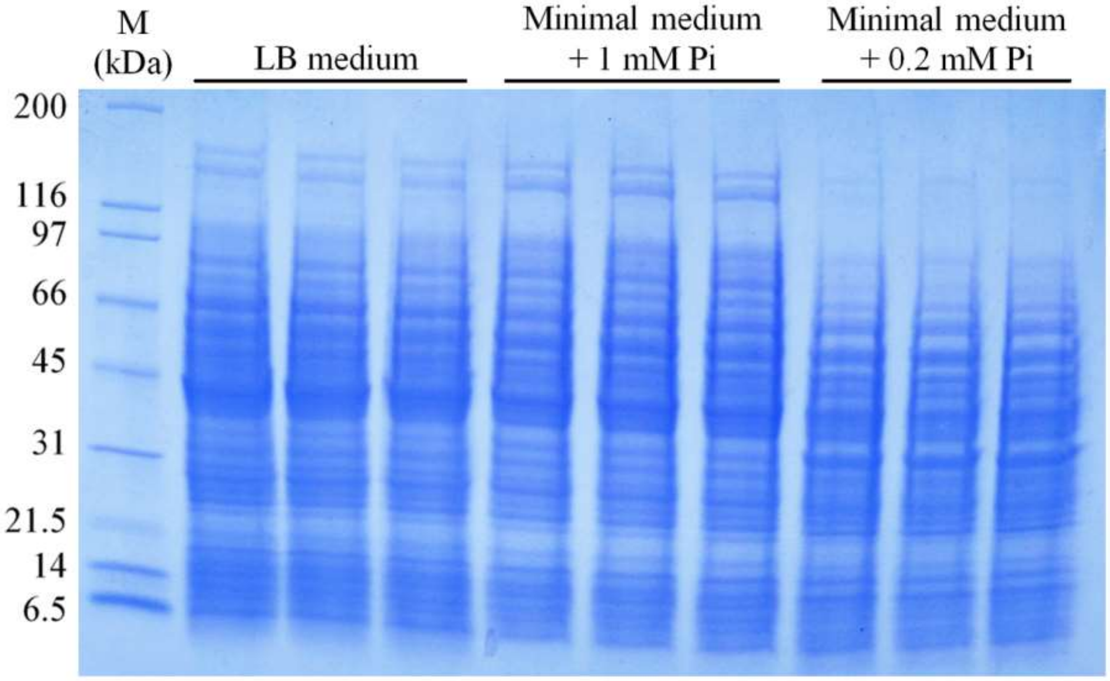

Figure S1. SDS-PAGE gel of the three replicates of protein extracts from *P. aeruginosa* PAO1 grown in LB, Minimal medium plus 0.2 mM Pi and Minimal medium plus 1 mM Pi. This gel was used for the proteomics analysis. Figure reproduced with permission from Matilla et al. (2022) Microbiol Spectr.: e0259022.

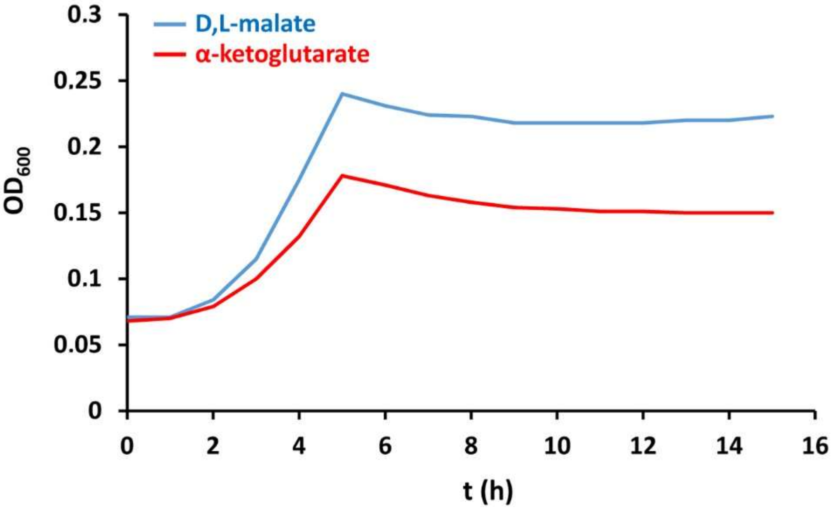

Figure S2. Growth experiments of *P. aeruginosa* PAO1 with D,L-malate and α-ketoglutarate as sole carbon sources. Growth in M9 minimal medium supplemented with 10 mM D,L-malate and α-ketoglutarate were conducted in 96-well plates. Cells were grown at 37 °C using a Bioscreen microbiological growth analyzer (Oy Growth Curves Ab Ltd., Helsinki, Finland) under continuous shaking.
